# Analysis of longitudinal patterns and predictors of medicine use in residential aged care using group‐based trajectory modelling: The MEDTRAC‐Polypharmacy longitudinal cohort study

**DOI:** 10.1111/bcp.16220

**Published:** 2024-08-25

**Authors:** Nasir Wabe, Rachel Urwin, Karla Seaman, Andrea Timothy, Magdalena Z. Raban, Johanna Westbrook

**Affiliations:** ^1^ Australian Institute of Health Innovation Macquarie University North Ryde New South Wales Australia

**Keywords:** GBTM, polypharmacy, quality indicator programs, residential aged care

## Abstract

**Aims:**

Polypharmacy serves as a quality indicator in residential aged care facilities (RACFs) due to concerns about inappropriate medication use. However, aggregated polypharmacy rates at a single time offer limited value. Longitudinal analysis of polypharmacy patterns provides valuable insights into identifying potential overuse of medicines. We aimed to determine long‐term trajectories of polypharmacy (≥9 medicines) and factors associated with each polypharmacy trajectory group.

**Methods:**

This was a longitudinal cohort study using electronic data from 30 RACFs in New South Wales, Australia. We conducted group‐based trajectory modelling to identify and characterize polypharmacy trajectories over 3 years. We evaluated the model fitness using the Bayesian Information Criterion, entropy (with a value of ≥0.8 considered ideal) and several other metrics.

**Results:**

The study included 2837 permanent residents (median age = 86 years, 61.7% female and 47.4% had dementia). We identified five polypharmacy trajectory groups: group 1 (no polypharmacy, 46.0%); group 2 (increasing polypharmacy, 9.4%); group 3 (decreasing polypharmacy, 9.2%); group 4 (increasing‐then decreasing polypharmacy, 10.0%), and group 5 (persistent polypharmacy, 25.4%). The model showed excellent performance (e.g., entropy = 0.9). Multinomial logistic regressions revealed the profile of each trajectory group (e.g., group 5 residents had higher odds of chronic respiratory disease compared with group 1).

**Conclusions:**

Our study identified five polypharmacy trajectory groups, including one with over a quarter of residents following a persistently high trajectory, signalling concerning medication overuse. Quality indicator programs should adopt tailored metrics to monitor diverse polypharmacy trajectory groups, moving beyond the current one‐size‐fits‐all approach and better capturing the evolving dynamics of residents' medication regimens.

**What is already known about this subject**

- Polypharmacy (the use of ≥9 medicines) is common among older people in residential aged care facilities (RACFs).
- Polypharmacy is commonly used as an indicator of the quality of aged care services, but its assessment relies on single‐time data, limiting its value in identifying risk factors or targeted intervention areas.

**What this study adds**

- Older adults in RACFs exhibit five distinct polypharmacy trajectories, including one group representing 25% of residents following persistently high polypharmacy.
- One in five residents display reduced polypharmacy, suggesting possible deprescribing practices.
- Each trajectory group exhibits distinctive baseline characteristics, providing a foundation for tailoring interventions to enhance medication use in RACFs.
- The study provides valuable insights that enhance quality indicator programs by enabling dynamic quality indicator reporting.

## 1 INTRODUCTION

As life expectancy increases, a greater number of older people are living with chronic health conditions that require treatment with medicines.^1^, ^2^, ^3^ This applies particularly to residential aged care facilities (RACFs), where residents are more likely to have multiple long‐term health conditions and complex care needs that are managed with multiple medicines.^4^ Suboptimal prescribing in RACFs increases the risk of experiencing medicine‐related harms such as adverse drug reactions, drug–drug interactions, increased risk of falls, unplanned hospitalization and premature death.^5^, ^6^, ^7^, ^8^

The concurrent use of multiple medicines, or polypharmacy,^1^, ^9^ is common among older people^10^, ^11^ and is more prevalent in RACFs (also referred to as long‐term care, care homes or nursing home) than in community‐dwelling settings.^12^ While there is no standard definition of polypharmacy,^6^, ^13^ it is most commonly defined in the literature as the concurrent use of five or more medicines.^1^, ^14^ Hyperpolypharmacy refers to the routine use of 10 or more medicines^15^, ^16^ as a way to distinguish it from polypharmacy, and is becoming increasingly common.^13^ Disparate definitions of polypharmacy present challenges when comparing polypharmacy prevalence between different settings and populations.^6^, ^11^, ^14^ A systematic review of 44 studies assessing medication use in long‐term care facilities reported polypharmacy prevalence of 38.1–91.2% for five or more medicines, 12.8–74.4% for nine or more medicines, and 10.6–65.0% for 10 or more medicines.^10^ Factors associated with polypharmacy in long‐term care facilities include recent hospital discharge,^17^ number of prescribers^18^ and multiple comorbidities.^19^, ^20^

While long‐term polypharmacy may reflect appropriate care in instances of complex multimorbidity,^21^ studies have reported associations between persistent polypharmacy in older people and increased fall injury risk, reduced physical function and reduced cognitive capability.^22^, ^23^, ^24^ As people age, the risk–benefit ratio of many medications changes, and hence regular medication reviews and the ceasing of specific medications are critical to effective medication management and improved patient outcomes.

Quality indicators are essential tools in healthcare, designed to measure various aspects of care and patient outcomes. They serve as benchmarks for evaluating the effectiveness, safety and efficiency of healthcare services.^25^ In Australia, where the performance of RACFs is monitored through the National Aged Care Mandatory Quality Indicator Program (NACMQIP), the polypharmacy quality indicator is defined as the prescription of nine or more medicines to a care recipient and is calculated by reviewing the medication charts or administration records for each care recipient on a selected collection date every quarter.^26^ Traditional quality indicators in polypharmacy often rely on single‐time aggregated data, providing a snapshot of medication use at a particular time. However, this approach may not capture the dynamic nature of medication use over time, potentially overlooking trends and patterns that could inform better management strategies. Longitudinal data provides a comprehensive understanding of polypharmacy by tracking medication use over time. It reveals trends such as initiation, discontinuation and modification of medications, identifying patterns that may correlate with adverse outcomes. This approach offers a nuanced view of medication management and provides valuable insights into the potential effectiveness of interventions, such as medication reviews and deprescribing programs, in addressing inappropriate polypharmacy.

Group‐based trajectory modelling (GBTM)^27^, ^28^ is a statistical method that has been applied in clinical research to group individuals with similar baseline characteristics and longitudinal patterns of change.^29^ Using this approach, patterns of medicine use and changes in polypharmacy status in older people have been used to group individuals according to their polypharmacy trajectories over time.^9^, ^30^ An Australian study using medication claims data from a population sample of Australians who were aged ≥70 identified two predominant polypharmacy trajectory groups in the cohort: sustained polypharmacy (77%, four trajectories) and decreasing medicine use (23%, three trajectories).^9^ In a study of older Australian women with and without dementia, the prevalence of polypharmacy increased over time and four distinct polypharmacy trajectories were identified: consistent polypharmacy; low polypharmacy; rapid increasing polypharmacy; and moderate polypharmacy.^30^ While polypharmacy is more prevalent in RACFs, compared to community‐dwelling older people,^6^, ^12^ there is limited information about polypharmacy trajectories among older people following their entry into RACFs. Such information may be particularly valuable in the RACF setting, providing an opportunity to implement targeted programs for enhancing medication management for residents. A critical advancement in this study is the use of unique medication administration data instead of relying on medication claims or dispensing records, unlike previous studies.^9^, ^30^ Medication claims and dispensing records often provide an incomplete picture, as they document only the medications that have been prescribed or dispensed, not necessarily those that have been consumed by patients. In contrast, medication administration data offers precise documentation of the medications actually received by each resident on a daily basis, ensuring more accurate polypharmacy data.

The aim of this study is threefold: (i) to identify polypharmacy trajectories during the first 3 years of permanent stays in RACFs using the GBTM approach; (ii) to determine factors associated with trajectory group membership; and (iii) to assess facility‐level variation in the rate of individuals exhibiting a persistent polypharmacy trajectory. This study is the first component of the MEDTRAC (MEDicine use Trajectories in Residential Aged Care) studies, conducted as part of the National Aged Care Medication Roundtable—a five‐year project funded by the Australian National Health and Medical Research Council.^31^

## 2 METHODS

### 2.1 Study setting

We conducted a retrospective longitudinal cohort study conducted using electronic aged care data extracted from 30 RACFs across two not‐for‐profit aged care providers (24 facilities from Provider A and six from Provider B) in the Sydney metropolitan region, New South Wales, Australia. The average facility sizes were 90 for Provider A and 110 for Provider B. Both providers offer holistic aged care, emphasizing quality of life through diverse social activities, comprehensive healthcare services and modern amenities. Their facilities provide tailored care options, including specialized dementia care, palliative care and respite care. Provider A also provides transition care and short‐term restorative care, while Provider B offers chronic and complex care and focuses mainly on older adults from migrant communities. Additionally, both providers offer Department of Veterans' Affairs (DVA)‐subsidized care and other government‐subsidized care for eligible adults. The study population from both providers consisted of older adults whose demographics aligned with typical Australian nursing home residents—a diverse ageing group with varying care needs and eligibility for different types of health services. Typically, the Australian nursing home population is two‐thirds female, with most residents aged 85 years or older, and about half having dementia.^32^ This demographic profile is consistent with the population in our study (Table 1). The study period was from 1 January 2019 to 30 September 2022. This study was reviewed and approved by the Macquarie University Human Research Ethics Committee (ref: 520231126749629).

**TABLE 1:** Baseline demographics and clinical characteristics.

Variables	*N* = 2837
Female	1751 (61.7)
Age in year, median (IQR)	86 (81–91)
Provider, *n* (%)	
A	2184 (78.0)
B	653 (23.0)
Year of admission, *n* (%)	
2019	773 (27.3)
2020	615 (21.7)
2021	823 (29.0)
2022 (Jan–Sep)	626 (22.1)
Health conditions, *n* (%)	
Circulatory conditions, any	2552 (90.0)
Cerebrovascular accident	691 (24.4)
Endocrine, any	1182 (41.7)
Diabetes	863 (30.4)
Thyroid	320 (11.3)
Chronic respiratory	571 (20.1)
Cancer	917(32.2)
Dementia	1344 (47.4)
Parkinson's disease	231 (8.14)
Depression, mood and affective disorders	1197 (42.2)
Anxiety and stress‐related disorders	830 (29.3)
Peptic ulcer and gastro‐oesophageal reflux disease	900 (31.7)
Renal disease	604 (21.3)
Arthritis	1528 (53.9)
Osteoporosis	802 (28.3)
Gout	218 (7.7)
Fracture	878 (31.0)
Hearing impairment	563 (19.8)
Visual impairment	431 (15.2)
Medications (ATC level 1), *n* (%)^a^	
Alimentary tract and metabolism	2514 (88.6)
Antineoplastic and immunomodulators	127 (4.5)
Blood and blood forming organs	1685 (59.4)
Cardiovascular system	2172 (76.6)
Genitourinary system and sex hormones	372 (13.1)
Musculoskeletal system	479 (169)
Nervous system	2274 (80.2)
Respiratory system	573 (20.2)
Sensory organs	708 (25.0)
Systemic hormonal preparations	632 (22.3)

^a^ Medication use at ATC level 1 with <2% usage was excluded.

### 2.2 Participants

The participants in the study were selected from a cohort of residents who were receiving care at one of the study facilities during the study period. The inclusion criteria were permanent residents aged 65 years or older who entered the RACFs on or after 1 January 2019 and stayed at the facility for at least 30 days to complete a minimum of four weekly follow‐up data points. We excluded residents who were already living in the RACF as of 1 January 2019, as we were interested in studying the progression of polypharmacy over time from the time of RACF entry. Temporary residents who were receiving respite or interim care were also excluded from the study as their stays were short‐term.

### 2.3 Data source

We utilized de‐identified routinely collected electronic health record (EHR) data provided by the two aged care providers. We linked two databases—*resident profile* and electronic Medication Administration Record (eMAR)—to obtain the relevant comprehensive demographic and clinical data. These datasets have been used in previous similar studies.^33^, ^34^, ^35^, ^36^, ^37^, ^38^ The datasets were collected as part of routine care provided by the aged care facility. The aged care providers were responsible for extracting and verifying the data to ensure its accuracy. Each dataset was then de‐identified and securely transferred to the research team. The *resident profile* database is part of the EHR system, designed to capture demographic and health conditions at the time of admission to the RACF. This included demographic information such as age, sex, the date and time of admission, and admission type, as well as information about the resident's baseline health conditions (e.g., whether they had dementia, Parkinson's disease or diabetes). The health conditions were a free‐text field which was processed using a previously developed health macro to identify health conditions.^39^ In this paper, we included conditions that were relevant to our study (Table 1) and had a prevalence of at least 5% at baseline.

The eMAR is a digital system used to manage and document the daily administration of medications to residents. It records details such as dosage, time, date and any related notes for each medication given. Widely implemented in Australian healthcare,^40^ eMAR has been shown to improve medication management.^41^, ^42^ We used the World Health Organization's Anatomical Therapeutic Classification (ATC)^43^ level five codes to identify unique administered medication in order to estimate polypharmacy. We counted each administered medication only once per day for each resident, regardless of the frequency or dose of administration. A medication combination consisting of multiple active ingredients in a single formulation (i.e., fixed‐dose combination) was considered a single medication for the purpose of determining polypharmacy, regardless of the number of active ingredients it contains.^44^ For example, N02BE71 (Zaldiar), a fixed‐dose combination of paracetamol and tramadol used for pain relief was counted as one medication. The ATC coding was performed by the research team (K.S.), based on the World Health Organization ATC database while also considering the local context.

### 2.4 Measures

Polypharmacy was defined as the administration of nine or more medicines to a resident on a given review date. This aligns with the Australian NACMQIP in terms of the number of medicines considered for defining polypharmacy.^26^ Our study used medication administration data records which provide precise information about the medicines that were taken by each resident on a given day, rather than prescription data, which summarizes all daily scheduled medicines and covers both administered and non‐administered medicines. Our primary analysis focused on *polypharmacy without medications taken as needed (PRN)*. Additionally, we reported polypharmacy that included all medicines, including PRN medications.

In alignment with the NACMQIP,^26^ the following short‐term medicines were excluded from medication counts for determining polypharmacy in this study: nutritional supplements, dermatologicals (ATC level 1 code D), anti‐infective for systemic use (ATC level 1 code J), gynaecological anti‐infectives and antiseptics (ATC level 2 code G01), anti‐infective preparations for ophthalmological use (ATC level 3 code S01A), anti‐infective preparations for otological use (ATC level 3 code S02A), anti‐infective preparations for use in eye or ear (ATC level 3 code S03A), intestinal anti‐infectives (ATC level 3 code A07A), anti‐infectives and antiseptics for local oral treatment (ATC level 4 code A01AB), combination medications for eradication of *Helicobacter pylori* (ATC level 4 code A02BD) and anti‐infectives and antiseptics for local oral treatment (ATC level 4 code R02AB).

### 2.5 Statistical analysis

#### 2.5.1 Group‐based trajectory modelling

The polypharmacy trajectory groups were identified using a GBTM approach^27^ with ‘traj’ package in Stata.^45^ We used a logistic model given polypharmacy status is a binary measure. GBTM is a type of finite mixture model used to identify distinct subgroups within a population that follow similar patterns of change (i.e., developmental trajectories) over time.^27^ The approach has been widely used in pharmacoepidemiologic studies in recent years.^9^, ^30^, ^46^, ^47^ We assessed the status of polypharmacy on a weekly basis over 3 years, starting from the resident's entry into the RACF, resulting in a total of 156 weekly polypharmacy values. GBTM assumes that data are missing at random, and utilizes available information to impute any missing polypharmacy values during the follow‐up time points.^28^, ^29^ It utilizes robust maximum likelihood estimation methods to derive unbiased parameter estimates.^28^

#### 2.5.2 GBTM model‐building process

The process of fitting GBTM models is iterative and requires specifying a predetermined number of groups to be examined, as well as determining the functional forms of each trajectory's evolution. We followed a two‐step procedure to determine the final polypharmacy GBTM model.

In step 1, we focused on determining the ideal number of trajectory groups. We fit models with 2–8 trajectory groups using the second‐order polynomials and applied the following set of criteria to identify the ideal number of trajectories. First, we selected the model with the lowest Bayesian Information Criterion (BIC) and Akaike Information Criterion (AIC) values. These model fit statistics provide a measure of how well the model fits the data, with lower values indicating better fit. Second, we set a minimum group size of 5% to ensure adequate sample size for statistical power. Third, we assessed the stability of the chosen number of groups across different random samples of the data. To test this, we implemented a 5‐fold cross‐validation approach by randomly splitting the data into five subsets and conducting a GBTM on each subset. We examined the consistency of the results in terms of both model fit statistics and group size information across the five subsets. This allowed us to confirm the robustness of our chosen number of groups and validate the reliability of our grouping approach.

Step 2 focused on determining the ideal shape of change (functional forms) of each trajectory. After the ideal number of trajectory groups was identified in step 1, all possible functional forms ranging from zero‐order (constant) to third‐order (cubic) polynomial were tested. The ideal function forms were selected based on model fit statistics, group size and cross‐validation results as outline in step 1.

#### 2.5.3 Evaluating final GBTM model fitness

After the final trajectory group numbers and their functional forms were determined, we utilized three measures including average posterior probability (APP), relative entropy and odds of correct classification (OCC) to evaluate model fit adequacy. The posterior probability indicates the likelihood of an individual belonging to a particular trajectory group, ranging from 0 to 1, with higher values indicating a better fit. An APP value of ≥0.7 in all groups is generally considered an acceptable minimum threshold.^28^, ^29^, ^48^ Relative entropy is a metric used to assess the quality of classification, with a range from 0 to 1, where higher values indicate better classification. A relative entropy of ≥0.8 implies a high level of certainty in the classification.^29^, ^48^ The OCC is calculated from the posterior probabilities and determines the predictive ability of group classification, with a value of ≥5 in all trajectory groups considered acceptable.^28^, ^29^, ^46^, ^48^ In GBTM research, once trajectory groups are identified, they can be labelled based on the shape or pattern of the depicted trajectories to capture defining characteristics and facilitate the interpretation of results.^49^ In our study, consistent with previous studies,^50^, ^51^ polypharmacy trajectory groups were named following a visual inspection of their dynamic evolution over time. Each group was labelled based on its distinctive pattern of development, considering both the level (e.g., no polypharmacy) and direction (e.g., increasing polypharmacy) of growth.

#### 2.5.4 Multinomial logistic regression

After identifying the trajectory groups, multinomial logistic regression was used to determine factors associated with trajectory group membership (objective 2). The selection of health conditions to assess their association with trajectory groups was made, considering their relevance to polypharmacy. This decision was supported by the existing literature, statistical significance and guided by clinical expertise. Relevant factors considered in the analyses were demographics (age, sex, provider), year of admission, baseline health status (Table 1), and baseline medication usage (ATC level 1). We determined the baseline medication usage status based on medication use during the first week of a resident's admission to the RACF.

We assessed multicollinearity among all potential pairs of baseline variables using correlation analysis, setting a >0.7 cut‐off for high correlation and selecting one variable in cases of high correlation.^52^ Next, we performed univariate analysis and selected variables with *P* ≤ .2 for the subsequent multivariate model. Multivariate multinomial regression with backward hierarchical selection was then used to identify factors independently associated with trajectory membership. The strength of association was estimated using a relative risk ratio (RRR) with 95% confidence interval (CI). RRR measures the likelihood of belonging to a specific trajectory group relative to the reference group, for every one‐unit increase in the predictor of interest, while keeping all other variables in the model constant.

##### Funnel plots

After identifying the trajectory groups, we used funnel plots to assess facility‐level variation in the rate of individuals following the problematic polypharmacy trajectory (i.e., persistent polypharmacy group). Funnel plots were generated by plotting each facility's risk‐adjusted rate in a scatterplot against its size, with 95% and 99.8% control limits superimposed around the overall rate.

All *P*‐values were two‐tailed, and we considered *P* < .05 to be statistically significant. The analysis was performed using Stata version 17 (StataCorp LP, College Station, TX).

## 3 RESULTS

### 3.1 Participants

A total of 2837 residents fulfilled the inclusion criteria. The median age of residents was 86 years (IQR 81–91), 61.7% were female and 47.4% had a dementia diagnosis at baseline. A summary of participant demographic and clinical characteristics is shown in Table 1.

### 3.2 Trends in overall polypharmacy rate

**FIGURE 1:**
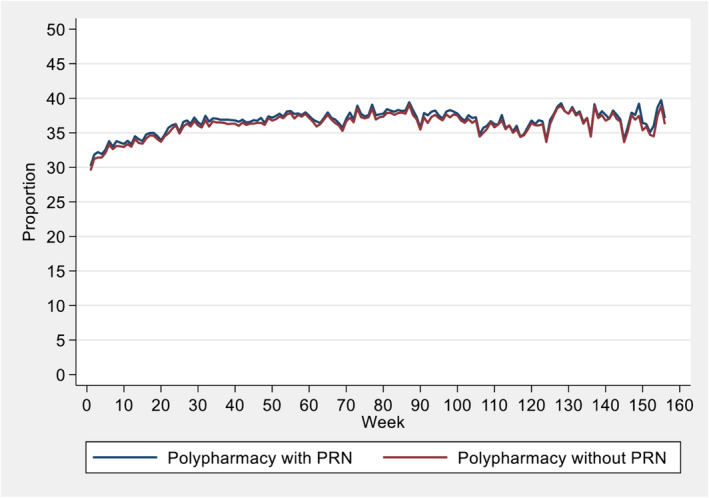
Proportion of polypharmacy during the first 3 years after entering RACFs. Polypharmacy was defined as using ≥9 medicines, with or without PRN medications.

Figure 1 presents the overall polypharmacy rate over a 3‐year period from admission to the RACFs. The overall polypharmacy rate increased over time, starting at approximately 30% at baseline, reaching 37% in the first 6 months, and then fluctuating between 34% and 40% for the remainder of the study period.

### 3.3 Polypharmacy trajectory groups

**FIGURE 2:**
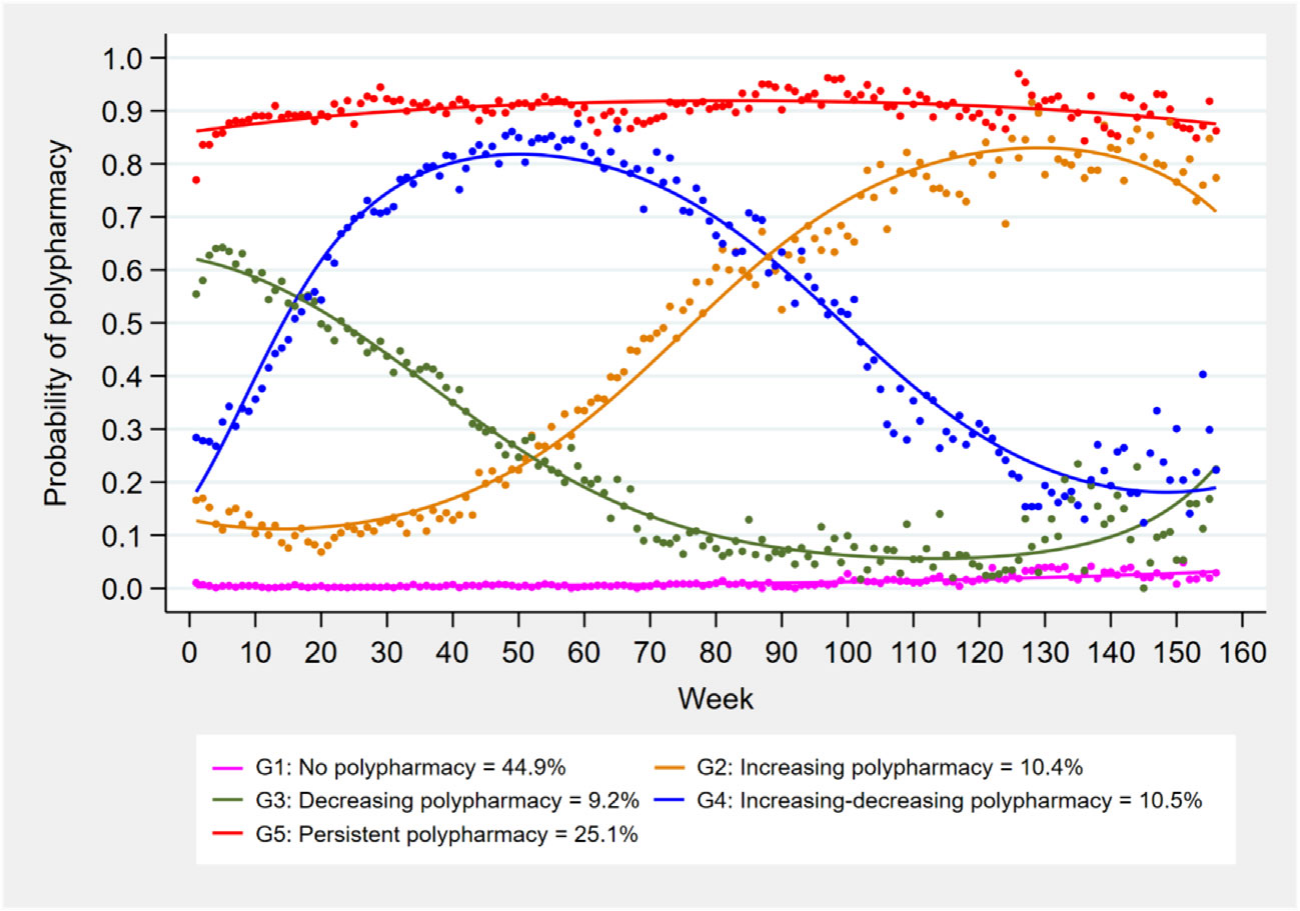
Polypharmacy trajectories during the first 3 years after RACF entry. GBTM was fit using a logit model. The solid lines indicate estimated probabilities, while the dots denote the actual probabilities. The legend indicates group size (%) derived from the estimated probabilities, which differ slightly from the observed group size presented in the text and in Tables S2 and S3.

A five‐group model was identified as the optimal number of polypharmacy trajectory groups. The identified five groups were:gGroup 1 (no polypharmacy, 46.0%, *n* = 1305); Group 2 (increasing polypharmacy, 9.4%, *n* = 268); Group 3 (decreasing polypharmacy, 9.2%, *n* = 260); Group 4 (increasing‐then decreasing polypharmacy, 10.0%, *n* = 284), and Group 5 (persistent polypharmacy, 25.4%, *n* = 720). The results indicate that 70% of residents (combining Groups 1 and 5) either never experience polypharmacy (Group 1) or consistently experience it (Group 5), while 30% of residents exhibit fluctuations in polypharmacy rates, showing either an increase or decrease over time. Two trajectory groups (Groups 3 and 4), with a combined 20% of residents, displayed decreasing levels of polypharmacy at some stage during the study period (Figure 2).

The median number of medications varied across trajectory groups from five (IQR 3–6) in Group 1 (no polypharmacy) to 11 (IQR 10–13) in Group 5 (persistent polypharmacy) (Figure S1). The GBTM model fit statistics for testing 2–8 trajectory groups are presented in Table S1. Model performance parameters are shown in Table S2. The model demonstrated excellent performance, with key metrics such as APP (≥0.88 for all groups, with a value of ≥0.7 considered acceptable), OCC (>40 for all groups with a value of ≥5 considered acceptable), and relative entropy of 0.9.

**FIGURE 3:**
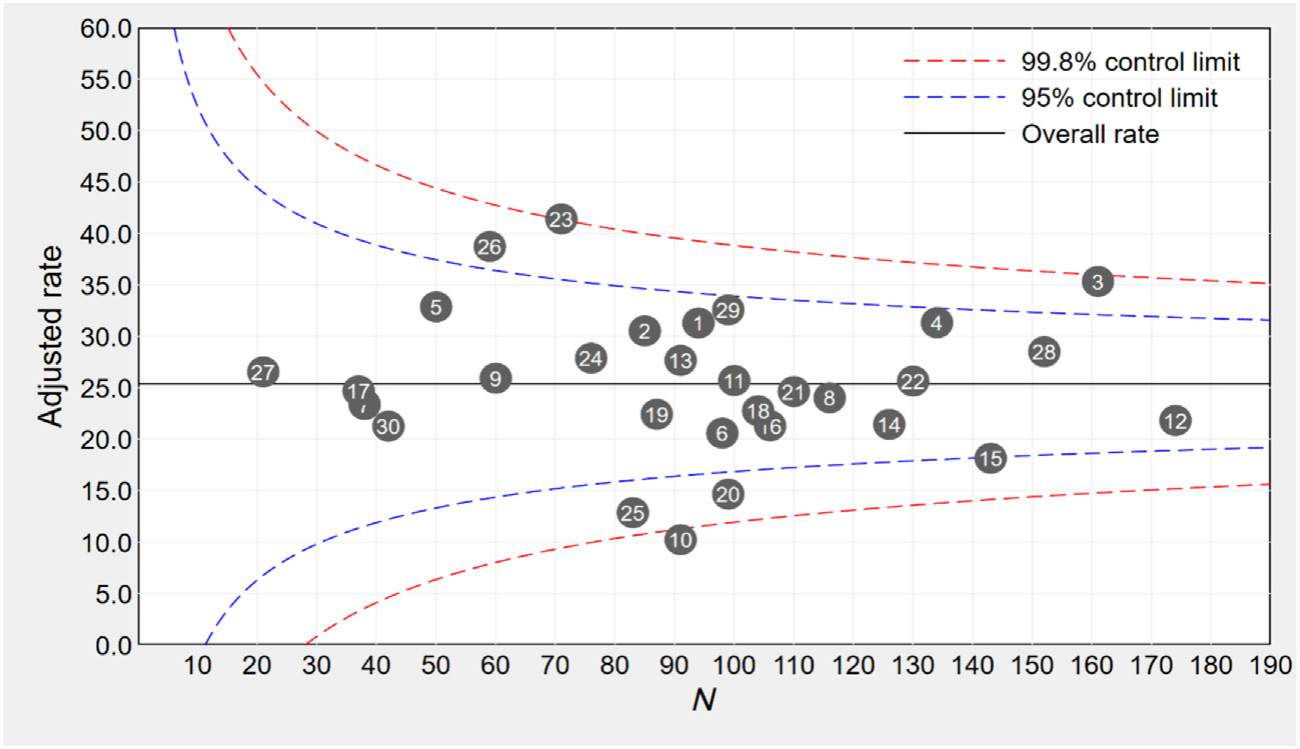
Funnel plot of the adjusted rates of persistent polypharmacy for residents in each RACF. The circles represent facilities, and the solid line represents the overall persistent polypharmacy rate. Rates were risk‐adjusted. Covariates used in risk adjustment included age, sex, dementia, osteoporosis, arthritis, stroke, Parkinson's disease and endocrine disorders.

Figure 3 presents a funnel plot illustrating variation in the rate of individuals following the persistent polypharmacy trajectory (Group 5) in each of the 30 facilities. Overall, although a quarter of individuals followed the persistent polypharmacy trajectory, there were substantial variations in adjusted rates of persistent polypharmacy across facilities. The adjusted rate ranged from 10.2% (facility #10) to 41.4% (facility #23). Three facilities (#3, #23 and #26) were outside the upper 95% control limit, suggesting “warning” for persistent polypharmacy.

### 3.4 Factors associated with polypharmacy trajectory membership

Table 2 presents results of multinomial logistic regressions showing baseline factors associated with polypharmacy trajectory membership using Group 5 (persistent polypharmacy) as a reference group. Gender and five health conditions (chronic respiratory disease, cancer, depression, mood and affective disorders, renal disease and visual impairment) were predictors of at least one trajectory group. For instance, for females compared to males, the relative risk of belonging to Group 3 (decreasing polypharmacy) over Group 5 (persistent polypharmacy) decreased by a factor of 0.67 after adjusting for variables in the model (RRR 0.67; 95% CI 0.47–0.94, *P* = .020). In simpler terms, females exhibited a higher likelihood than males of being in Group 5 as opposed to Group 3. Individuals having renal disease at baseline were more likely to be in Group 4 (increasing‐then decreasing polypharmacy) *vs*. Group 5 (RRR 1.39, 95% CI 1.04–1.85, *P* = .027).

**TABLE 2:** Multinomial logistic regressions showing factors associated with polypharmacy trajectory membership using group 5 (persistent polypharmacy) was used as the reference category. *P* < .05 indicated in bold.

	Group 1: no polypharmacy		Group 2: increasing polypharmacy		Group 3: decreasing polypharmacy		Group 4: increasing‐then decreasing	
	RRR (95% CI)	P	RRR (95% CI)	P	RRR (95% CI)	P	RRR (95% CI)	P
Female *vs*. Male	0.94 (0.73–1.20)	0.618	0.92 (0.68–1.24)	0.572	**0.67 (0.47–0.94)**	0.020	1.25 (0.92–1.70)	0.160
Age in years	1.01 (0.99–1.03)	0.158	0.99 (0.97–1.02)	0.627	0.99 (0.97–1.02)	0.594	1.01 (0.99–1.02)	0.528
***Health conditions***								
Provider B *vs*. Provider A	1.05 (0.76–1.46)	0.755	1.01 (0.76–1.34)	0.929	0.74 (0.49–1.13)	0.164	1.19 (0.76–1.85)	0.454
Any circulatory conditions	0.68 (0.43–1.09)	0.109	1.17 (0.56–2.45)	0.674	1.16 (0.66–2.05)	0.604	1.03 (0.61–1.72)	0.915
Any endocrine conditions	0.72 (0.51–1.02)	0.067	1.34 (0.95–1.88)	0.099	1.51 (0.99–2.32)	0.058	1.08 (0.69–1.67)	0.747
Chronic respiratory disease	**0.65 (0.44–0.96)**	0.029	0.70 (0.46–1.06)	0.095	1.18 (0.76–1.83)	0.468	0.93 (0.59–1.45)	0.740
Cancer	**1.31 (1.01–1.71)**	0.042	1.17 (0.89–1.54)	0.270	0.91 (0.60–1.36)	0.630	1.28 (1.00–1.65)	0.052
Parkinson's disease	1.26 (0.47–3.42)	0.643	2.03 (0.78–5.31)	0.149	2.85 (1.08–7.54)	0.035	2.19 (0.76–6.28)	0.144
PUD/GORD^a^	0.90 (0.69–1.18)	0.441	1.17 (0.83–1.64)	0.370	0.89 (0.67–1.18)	0.423	1.05 (0.81–1.37)	0.685
Renal disease	1.17 (0.86–1.59)	0.307	1.35 (0.96–1.92)	0.086	1.27 (0.96–1.69)	0.095	**1.39 (1.04–1.85)**	0.027
Dementia	1.03 (0.77–1.38)	0.827	0.78 (0.55–1.11)	0.168	0.95 (0.71–1.28)	0.734	0.96 (0.77–1.19	0.689
Arthritis	0.87 (0.70–1.08)	0.212	1.00 (0.73–1.36)	0.982	1.20 (0.91–1.58)	0.197	0.99 (0.76–1.28)	0.920
Gout	0.92 (0.53–1.59)	0.764	0.69 (0.32–1.51)	0.356	0.91 (0.43–1.91)	0.798	1.23 (0.70–2.18)	0.471
Fracture	0.87 (0.73–1.04)	0.133	1.09 (0.82–1.46)	0.550	1.14 (0.85–1.53)	0.392	0.92 (0.66–1.27)	0.602
Cerebrovascular accident	1.18 (0.81–1.71)	0.393	0.96 (0.63–1.47)	0.857	0.91 (0.62–1.35)	0.641	0.86 (0.63–1.19)	0.364
Depression, mood and affective disorders	**0.74 (0.56–0.99)**	0.042	1.21 (0.87–1.69)	0.258	1.11 (0.78–1.59)	0.551	1.32 (0.89–1.95)	0.170
Anxiety and stress‐related disorders	0.91 (0.71–1.16)	0.454	1.11 (0.81–1.52)	0.524	1.10 (0.74–1.64)	0.622	0.93 (0.65–1.32)	0.677
Visual impairment	**0.75 (0.58–0.96)**	0.022	0.92 (0.63–1.35)	0.666	1.25 (0.89–1.77)	0.198	1.09 (0.70–1.70)	0.699
***Medications (ATC level 2)***								
Renin‐angiotensin‐system‐acting agents	**0.41 (0.31–0.53)**	0.000	**0.57 (0.43–0.75)**	0.000	0.79 (0.61–1.03)	0.085	**0.74 (0.57–0.97)**	0.029
Analgesics	**0.58 (0.44–0.77)**	0.000	1.12 (0.77–1.62)	0.567	1.02 (0.75–1.38)	0.897	1.23 (0.84–1.79)	0.293
Anti‐Parkinson's drugs	**0.25 (0.09–0.730**	0.011	**0.28 (0.10–0.73)**	0.009	**0.26 (0.09–0.75)**	0.012	0.35 (0.12–1.03)	0.057
Antianemic preparations	**0.42 (0.31–0.58)**	0.000	0.65 (0.42–1.01)	0.054	0.92 (0.63–1.32)	0.637	0.97 (0.67–1.41)	0.890
Antiepileptics	**0.29 (0.22–0.39)**	0.000	**0.60 (0.40–0.90)**	0.013	0.99 (0.73–1.35)	0.972	**0.74 (0.55–0.99)**	0.042
Antigout preparations	**0.50 (0.22–1.09)**	0.082	1.14 (0.47–2.79)	0.776	0.98 (0.39–2.46)	0.966	0.85 (0.44–1.66)	0.640
Antithrombotic agents	**0.40 (0.29–0.55)**	0.000	**0.66 (0.45–0.97)**	0.034	0.78 (0.56–1.09)	0.143	**0.62 (0.45–0.87)**	0.005
Beta blocking agents	**0.53 (0.40–0.70)**	0.000	**0.55 (0.39–0.78)**	0.001	0.81 (0.57–1.16)	0.248	0.90 (0.68–1.19)	0.455
Calcium channel blockers	**0.44 (0.31–0.62)**	0.000	**0.61 (0.46–0.82)**	0.001	1.03 (0.76–1.39)	0.849	0.86 (0.60–1.24)	0.420
Cardiac therapy	**0.29 (0.21–0.39)**	0.000	**0.61 (0.45–0.84)**	0.002	**0.69 (0.51–0.94)**	0.018	0.79 (0.58–1.07)	0.128
Corticosteroids for systemic use	**0.48 (0.28–0.83)**	0.008	0.92 (0.52–1.62)	0.777	0.66 (0.42–1.04)	0.072	0.78 (0.55–1.10)	0.159
Diuretics	**0.33 (0.24–0.46)**	0.000	**0.52 (0.37–0.73)**	0.000	**0.64 (0.45–0.90)**	0.012	**0.59 (0.41–0.85)**	0.005
Drugs for acid‐related disorders	**0.38 (0.29–0.50)**	0.000	**0.55 (0.41–0.73)**	0.000	1.08 (0.76–1.55)	0.659	0.75 (0.56–1.00)	0.054
Drugs for constipation	**0.41 (0.31–0.54)**	0.000	**0.62 (0.46–0.83)**	0.001	1.16 (0.81–1.65)	0.424	0.74 (0.54–1.03)	0.077
Drugs for obstructive airway diseases	**0.37 (0.22–0.63)**	0.000	0.67 (0.36–1.24)	0.206	**0.67 (0.46–0.97)**	0.036	0.83 (0.52–1.34)	0.450
Drugs used in diabetes	0.67 (0.41–1.11)	0.119	1.20 (0.76–1.87)	0.433	1.14 (0.73–1.78)	0.579	1.36 (0.93–1.99)	0.112
Lipid‐modifying agents	**0.36 (0.25–0.50)**	0.000	**0.54 (0.39–0.73)**	0.000	0.71 (0.51–0.98)	0.036	0.77 (0.54–1.09)	0.137
Mineral supplements	**0.37 (0.29–0.46)**	0.000	**0.57 (0.43–0.77)**	0.000	1.02 (0.77–1.37)	0.869	**0.57 (0.39–0.82)**	0.003
Ophthalmologicals	**0.28 (0.21–0.37)**	0.000	**0.53 (0.37–0.76)**	0.001	0.86 (0.64–1.16)	0.338	**0.70 (0.53–0.91)**	0.009
Psychoanaleptics	**0.51 (0.37–0.70)**	0.000	**0.56 (0.42–0.74)**	0.000	0.91 (0.68–1.21)	0.515	0.84 (0.64–1.11)	0.231
Psycholeptics	**0.31 (0.23–0.42)**	0.000	**0.45 (0.32–0.63)**	0.000	**0.67 (0.48–0.93)**	0.019	**0.49 (0.34–0.69)**	0.000
Thyroid therapy	**0.66 (0.43–1.00)**	0.048	**0.57 (0.34–0.96)**	0.033	0.91 (0.61–1.36)	0.649	0.85 (0.53–1.36)	0.489
Urologicals	**0.32 (0.23–0.45)**	0.000	**0.52 (0.33–0.81)**	0.004	0.72 (0.49–1.06)	0.097	0.63 (0.36–1.07)	0.088
Vitamins	**0.36 (0.26–0.50)**	0.000	**0.45 (0.30–0.67)**	0.000	**0.60 (0.44–0.82)**	0.001	**0.61 (0.44–0.85)**	0.003

^a^ Peptic ulcer disease and gastro‐oesophageal reflux disease.

As anticipated, baseline medication‐related variables exhibited significant differences across trajectory groups. Notably, individuals in Group 1 (no polypharmacy) were less likely to be on all medication classes, except for drugs used in diabetes, compared to those in Group 5 (Table 2). Additional analysis comparing all trajectory groups against Group 1 are presented in Table S3.

## 4 DISCUSSION

This study is the first to explore polypharmacy trajectories over a long follow‐up period in RACFs. Employing a longitudinal design with multiple polypharmacy assessments, we utilized GBTM methodology—a novel statistical approach—to characterize polypharmacy status over 3 years. We identified five trajectory groups with distinct polypharmacy characteristics. Notably, one trajectory group comprised over a quarter of residents (25.4%), following a persistently high polypharmacy trajectory, indicating a potential overuse of medicines. Gender, five health conditions (e.g., chronic respiratory disease, renal disease) and several baseline medication‐related variables emerged as predictors for at least one trajectory group. These findings serve as a crucial initial step towards identifying opportunities for early interventions to improve medication use in RACFs.

In this study, 54% of all residents, experienced polypharmacy at least once during the study period (Groups 2–5), while 46% of residents in Group 1 did not experience polypharmacy over the 3‐year period. Due to the absence of comparable studies utilizing similar methodology in RACFs, we are unable to make direct comparisons with our findings. Existing studies primarily report the point prevalence of polypharmacy using cross‐sectional data and employ various cut‐off points to define polypharmacy, such as 5, 9 and 10 medications, depending on the healthcare setting.^6^, ^10^, ^12^, ^53^, ^54^ A systematic review of 44 studies assessing medication use in RACFs reported polypharmacy prevalence of 12.8–74.4% for nine or more medications.^10^ The same review reported that a high prevalence of up to 91% was reported when polypharmacy was defined as the use of five or more medicines within this population.^10^ However, two prior Australian studies employed a similar methodology to determine polypharmacy trajectories, utilizing data from the Australian Pharmaceutical Benefits Scheme (PBS), but they did not specifically focus on RACFs.^9^, ^30^ Thapaliya et al. investigated polypharmacy trajectories in older women, with and without dementia, using medication claims data from 2003 to 2015 utilizing yearly indicators for polypharmacy (≥5 prescribed medicines). Their approach ignored any changes in polypharmacy status that may have occurred during a 12‐month window.^30^ That study identified four polypharmacy trajectory groups (low polypharmacy, moderate polypharmacy, consistent polypharmacy and rapidly increasing polypharmacy).^30^ Falster et al. explored polypharmacy trajectories among a population sample of Australians who were aged 70 or above.^9^ Polypharmacy (≥5 prescribed medicines) was determined at the midpoint of each subsequent quarter across the years 2014–2018.^9^ Trajectory modelling identified two groups: sustained polypharmacy (76.8%,); and declining use of medicines (23.2%); however, polypharmacy prevalence in the target population was not determined as only those who experienced polypharmacy at baseline were included in the study.^9^

In our study, we identified several factors associated with polypharmacy trajectory membership (e.g., chronic respiratory conditions, depression, being female). While direct comparisons are challenging, some prior studies have also reported factors associated with polypharmacy in different settings. For instance, a Biobank study found a higher prevalence of polypharmacy in patients with chronic obstructive pulmonary disease (COPD) compared to those without COPD.^55^ Chronic respiratory conditions, such as COPD, affect approximately one‐third of Australians,^56^ and medicine use among these patients was often inconsistent with international guidelines.^57^ A nationwide longitudinal study of polypharmacy among older adults in Sweden reported that older age, being female, multiple morbidities and living in an institution were associated with chronic polypharmacy.^58^ Additionally, an association between polypharmacy and depression among older adults has been reported previously.^59^, ^60^, ^61^ Interestingly, in our analysis, we did not observe a significant association between dementia status and trajectory group membership despite existing literature suggesting otherwise.^62^ This finding may be attributed to the characteristics of our study population or the inclusion of multiple variables, including several central nervous system medications, which could potentially offset the impact of dementia. However, our findings indicate a disparity that warrants further investigation in future research.

While the focus of the current study was not on medication appropriateness, the findings allude to potential inappropriate overuse of medicines, particularly among residents within persistent polypharmacy trajectory groups. Our findings reveal significant variation in persistent polypharmacy rates across the 30 RACF, ranging from 10.2% to 41.4%, even after adjusting for case‐mix. A cross‐sectional analysis of 589 long‐term care homes in Ontario, Canada, also reported wide variation in polypharmacy (≥9 medicines) rates across homes, from 7.9% to 26.2%, and proposed a role for facility‐level medication reviews to identify inappropriate prescribing.^20^ The study of polypharmacy trajectories among older Australian women with and without dementia reported that the likelihood of experiencing polypharmacy in a given year was higher among women without a medication review, compared to women who received medication review services in that year,^30^ highlighting the importance of strategies that identify medication‐related problems and inappropriate polypharmacy among older adults with complex health needs and comorbidities.

Our study identified one in five residents (20%) displaying decreasing levels of polypharmacy at some stage during their stay. This hints at potential deprescribing practices within the facilities. Deprescribing—withdrawing inappropriate medications under supervision—has a critical role in reducing potentially inappropriate medications and improving health outcomes.^63^, ^64^, ^65^ Nevertheless, further research is imperative to explore the specific medication classes contributing to this trend and to explore the factors associated with deprescribing in RACFs. Additionally, an examination of external factors linked to each trajectory group, including organizational structure, clinician‐related factors and socioeconomic status, is essential for a comprehensive understanding.

### 4.1 Implications for practice and policy

The findings of this study have important implications for national quality indicator programs and quality improvement initiatives within RACFs. GBTM can provide valuable insights that enhance quality indicator programs such as the Australian NACMQI^26^ by enabling dynamic quality indicator reporting. Quality indicator programs can leverage GBTM to develop more relevant metrics for evaluating and reporting polypharmacy. Instead of the current one‐size‐fits‐all approach of reporting polypharmacy rates at a single point in time, programs can develop indicators that reflect diverse polypharmacy trajectory groups, which track medication use over time and capture the evolving dynamics of residents' medication regimens. This methodology facilitates the identification of gradual increases or decreases in medication use, as well as any abrupt changes, providing a comprehensive view of medication management practices in RACFs. Nevertheless, the successful implementation of polypharmacy trajectory groups for dynamic quality indicator reporting relies on two pivotal factors. First, it necessitates the presence of well‐integrated electronic databases ensuring seamless data accessibility, covering electronic medication administration and patient demographic information. Second, it requires robust technological infrastructure, including advanced data analytics systems or software for identifying polypharmacy trajectory groups.

Importantly, incorporating polypharmacy trajectory group data into quality improvement programs allows for targeted initiatives, such as medication reviews, deprescribing initiatives and enhanced monitoring, aimed at reducing unnecessary polypharmacy. The foundational step in implementing this strategy lies in understanding the baseline profiles of the trajectory groups, which is crucial for facilitating early intervention to improve medication use and reduce problematic polypharmacy trajectories. In our study, 25.4% of residents were assigned to the persistent polypharmacy group, where the median number of administered medicines was 11 (range 9–22). This group possesses distinct baseline characteristics that differentiate them from other groups. For instance, they are more likely to be female compared to those in Group 3 (decreasing polypharmacy). Additionally, they exhibit a lower likelihood of having a cancer diagnosis but a higher prevalence of depression, mood and affective disorders, chronic respiratory disease and visual impairment at baseline compared to those in Group 1 (no polypharmacy). These specific characteristics at the time of entry into RACFs offer valuable insights for aged care providers to tailor interventions, addressing the unique needs and risks associated with each trajectory group. A potential early intervention strategy could involve tailoring Residential Medication Management Reviews (RMMRs)^66^ and other quality use of medicine services based on this profile (e.g., providing regular RMMRs for individuals who are likely to follow a persistent trajectory group), aiming to minimize inappropriate polypharmacy and enhance overall health outcomes in RACFs.

### 4.2 Strengths and limitations of the study

This is the first study to examine polypharmacy trajectories among older people following their entry into an RACF. A key strength of the study is the use of medication administration data rather than medication claims data. These data provide more accurate documentation of the medicines received by each resident and allowed us to estimate polypharmacy in relatively small windows of time (i.e., weekly). A limitation of the study is that the included RACFs were predominantly located within one metropolitan area in a single country, potentially limiting the generalizability and applicability of our findings to other contexts. While we accounted for several resident characteristics in our modelling to control for confounders, other potentially valuable variables, such as system‐related factors including variations in quality of care among different facilities, access to and implementation of quality improvement initiatives (e.g., medication reviews), as well as temporal biases across facilities (e.g., changes in prescribing practices over time), may influence polypharmacy trajectories. We recognize that not all cases of polypharmacy are inappropriate and may be justified in certain individuals. While our study identified various polypharmacy trajectory groups, which could imply potential overuse of medications in specific groups, the study did not assess the appropriateness of medication use. Other studies have reported an association between polypharmacy and potentially inappropriate use of medicines, although not in the context of trajectory modelling.^67^, ^68^, ^69^ Further research is warranted to investigate potentially inappropriate medication use and its influence on polypharmacy trajectories.

## 5 CONCLUSION

Our study identified five polypharmacy trajectory groups, including one with over a quarter of residents following a persistently high trajectory, signalling concerning medication overuse. We identified the baseline profile of polypharmacy trajectory groups, which can serve as a crucial initial step towards identifying opportunities for early interventions to improve medication use in RACFs. Quality indicator programs should adopt tailored metrics to monitor diverse polypharmacy trajectory groups over time, moving beyond the current one‐size‐fits‐all approach and better capturing the evolving dynamics of residents' medication regimens.

## AUTHOR CONTRIBUTIONS

N.W. designed the study, conducted the analysis, and drafted and revised the manuscript. R.U. and K.S. interpreted results, and drafted and revised the manuscript. A.T., M.Z.R. and J.W. reviewed results and drafted manuscript. All authors reviewed the final manuscript before submission.

## CONFLICT OF INTEREST STATEMENT

There are no competing interests to declare.

## Supporting information


**Table S1:** Model selection and performance using second‐order (quadratic) polynomials across trajectories.
**Table S2:** The performance of a 5‐group GBTM model using polynomials of orders 1, 3, 3, 3 and 2 across trajectory groups.
**Table S3:** Multinomial logistic regressions showing factors associated with polypharmacy trajectory membership using group 1 (no polypharmacy) as a reference.
**Figure S1:** Median number of medications by trajectory groups: 5 (IQR 3–6; range 0–8) for group 1; 7 (IQR 6–8; range 1–15) for group 2; 8 (IQR 7–9; range 1–17) for group 3; 9 (IQR 8–10; range 1–20) for group 4 and 11 IQR 10–13; range 9–22) for group 5.

## Data Availability

The data that support the findings of this study are available on request from the corresponding author. The data are not publicly available due to privacy or ethical restrictions.
