# Supplementary material for: Analysis of longitudinal patterns and predictors of medicine use in residential aged care using group‐based trajectory modelling: The MEDTRAC‐Polypharmacy longitudinal cohort study
Source: Br J Clin Pharmacol. 2024 Aug 25;90(12):3308–19. doi: 10.1111/bcp.16220 (PMC11602946; doi:10.1111/bcp.16220)
Supplement: Supplementary file 1 — Table S1: Model selection and performance using second‐order (quadratic) polynomials across trajectories. Table S2: The performance of a 5‐group GBTM model using polynomials of orders 1, 3, 3, 3 and 2 across trajectory groups. Table S3: Multinomial logistic regressions showing factors associated with polypharmacy trajectory membership using group 1 (no polypharmacy) as a reference. Figure S1: Median number of medications by trajectory groups: 5 (IQR 3–6; range 0–8) for group 1; 7 (IQR 6–8; range 1–15) for group 2; 8 (IQR 7–9; range 1–17) for group 3; 9 (IQR 8–10; range 1–20) for group 4 and 11 IQR 10–13; range 9–22) for group 5. [file BCP-90-3308-s001.docx]

Supplementary File

**Supplementary Table 1: Model selection and performance using second-order (quadratic) polynomials across trajectories**.

| **No. of**  **groups** | **BIC** | **AIC** | **>5% per**  **group** | **CV criteria fulfilled** | **APP per group >0.7** | **Relative**  **Entropy** | **>5 OCC**  **per group** |
| --- | --- | --- | --- | --- | --- | --- | --- |
|  |  |  |  |  |  |  |  |
| 2 | -65318.5 | -65297.7 | Yes | Yes | Yes | 0.98 | Yes |
| 3 | -56996.6 | -56963.9 | Yes | Yes | Yes | 0.94 | Yes |
| 4 | -53790.0 | -53745.3 | Yes | Yes | Yes | 0.89 | Yes |
| 5 | -51770.4 | -51713.9 | Yes | Yes | Yes | 0.90 | Yes |
| 6 | -50500.3 | -50431.8 | Yes | No | Yes | 0.85 | Yes |
| 7 | -49660.5 | -49580.1 | No | No | Yes | 0.84 | Yes |
| 8 | -49189.2 | -49096.9 | No | No | Yes | 0.82 | Yes |

BIC, Bayesian Information Criterion; AIC, Akaike Information Criterion; CV, Cross-Validation; APP, Average Posterior Probability; OCC, Odds of Correct Classification.

**Supplementary Table 2: The performance of a 5-group GBTM model using polynomials of orders 1, 3, 3, 3, and 2 across trajectory groups**

| **Groups** | **Group size, N** | **Proportion, maxPP^1^** | **Proportion, TotalPP^2^** | **APP** | **OCC** |
| --- | --- | --- | --- | --- | --- |
|  |  |  |  |  |  |
| 1 | 1,305 | 46.0 | 44.9 | 0.97 | 40.7 |
| 2 | 268 | 9.4 | 10.4 | 0.93 | 112.5 |
| 3 | 260 | 9.2 | 9.2 | 0.88 | 69.4 |
| 4 | 284 | 10.0 | 10.5 | 0.91 | 90.7 |
| 5 | 720 | 25.4 | 25.1 | 0.97 | 84.3 |
| BIC = -51693.9; AIC = -51631.4; Relative entropy = 0.90 | | | | | |

^1^Based on maximum posterior probability; ^2^Based on the sums of the posterior probabilities.

**Supplementary Table 3:** **Multinomial logistic regressions showing factors associated with polypharmacy trajectory membership using group 1 (no polypharmacy) as a reference.**

|  | **Group 2:**  **Increasing polypharmacy** | | **Group 3:**  **Decreasing** **polypharmacy** | | **Group 4:**  **Increasing-then decreasing** | | **Group 5:**  **Persistent polypharmacy** | |
| --- | --- | --- | --- | --- | --- | --- | --- | --- |
|  | **RRR (95% CI)** | **P** | **RRR (95% CI)** | **P** | **RRR (95% CI)** | **P** | **RRR (95% CI)** | **P** |
| Female vs Male | 0.98 (0.66-1.44) | 0.902 | 0.71 (0.47-1.06) | 0.093 | 1.33 (0.94-1.88) | 0.108 | 1.07 (0.83-1.36) | 0.618 |
| Age in year | 0.98 (0.96-1.00) | 0.068 | 0.98 (0.96-1.00) | 0.069 | 0.99 (0.97-1.01) | 0.431 | 0.99 (0.97-1.01) | 0.158 |
| Provider B vs Provider A | 0.96 (0.75-1.24) | 0.762 | 0.70 (0.47-1.07) | 0.098 | 1.13 (0.80-1.58) | 0.498 | 0.95 (0.69-1.31) | 0.755 |
| Any circulatory conditions | 1.71 (0.78-3.76) | 0.179 | 1.70 (0.97-2.97) | 0.063 | 1.50 (0.84-2.70) | 0.171 | 1.46 (0.92-2.33) | 0.109 |
| Any endocrine conditions | **1.85 (1.36-2.51)** | 0.000 | **2.10 (1.37-3.20)** | 0.001 | 1.49 (0.96-2.31) | 0.075 | 1.39 (0.98-1.96) | 0.067 |
| Chronic respiratory disease | 1.08 (0.75-1.56) | 0.691 | **1.81 (1.10-2.98)** | 0.019 | 1.43 (0.86-2.36) | 0.166 | **1.54 (1.05-2.27)** | 0.029 |
| Cancer | 0.89 (0.63-1.25) | 0.503 | **0.69 (0.51-0.94)** | 0.018 | 0.98 (0.71-1.34) | 0.880 | **0.76 (0.59-0.99)** | 0.042 |
| Parkinson’s disease | 1.61 (0.58-4.48) | 0.366 | 2.25 (0.67-7.59) | 0.191 | 1.73 (0.66-4.57) | 0.266 | 0.79 (0.29-2.14) | 0.643 |
| PUD/GORD* | 1.30 (0.91-1.84) | 0.146 | 0.99 (0.72-1.37) | 0.952 | 1.17 (0.93-1.47) | 0.173 | 1.11 (0.85-1.45) | 0.441 |
| Renal disease | 1.15 (0.85-1.56) | 0.351 | 1.09 (0.83-1.42) | 0.550 | 1.18 (0.88-1.59) | 0.273 | 0.85 (0.63-1.16) | 0.307 |
| Dementia | 0.76 (0.55-1.04) | 0.082 | 0.92 (0.68-1.24) | 0.582 | 0.93 (0.69-1.25) | 0.616 | 0.97 (0.73-1.29) | 0.827 |
| Arthritis | 1.15 (0.88-1.50) | 0.314 | 1.38 (0.98-1.94) | 0.064 | 1.13 (0.85-1.51) | 0.389 | 1.15 (0.92-1.43) | 0.212 |
| Gout | 0.75 (0.37-1.54) | 0.439 | 0.99 (0.51-1.90) | 0.967 | 1.34 (0.73-2.46) | 0.342 | 1.09 (0.63-1.88) | 0.764 |
| Fracture | 1.25 (0.92-1.70) | 0.147 | 1.31 (0.96-1.78) | 0.091 | 1.05 (0.80-1.39) | 0.719 | 1.15 (0.96-1.38) | 0.133 |
| Cerebrovascular accident | 0.82 (0.58-1.14) | 0.239 | 0.77 (0.53-1.14) | 0.190 | 0.73 (0.47-1.13) | 0.161 | 0.85 (0.58-1.24) | 0.393 |
| Depression, mood & affective disorders | **1.63 (1.10-2.42)** | **0.015** | **1.50 (1.12-2.02)** | **0.007** | **1.78 (1.16-2.73)** | **0.009** | **1.35 (1.01-1.80)** | **0.042** |
| Anxiety & stress-related disorders | 1.22 (0.89-1.66) | 0.217 | 1.21 (0.85-1.74) | 0.294 | 1.02 (0.73-1.42) | 0.915 | 1.10 (0.86-1.40) | 0.454 |
| Visual impairment | 1.23 (0.79-1.92) | 0.357 | **1.68 (1.19-2.38)** | 0.004 | 1.46 (0.94-2.27) | 0.092 | **1.34 (1.04-1.72)** | 0.022 |
| Renin-angiotensin-system-acting agents | 1.40 (0.99-1.96) | 0.054 | **1.95 (1.40-2.71)** | 0.000 | **1.83 (1.33-2.51)** | 0.000 | **2.46 (1.88-3.20)** | 0.000 |
| Analgesics | **1.92 (1.51-2.46)** | 0.000 | **1.76 (1.32-2.34)** | 0.000 | **2.11 (1.46-3.06)** | 0.000 | **1.72 (1.30-2.29)** | 0.000 |
| Anti-parkinson drugs | 1.09 (0.38-3.13) | 0.870 | 1.05 (0.27-4.11) | 0.944 | 1.41 (0.52-3.80) | 0.499 | **3.97 (1.38-11.43)** | 0.011 |
| Antianemic preparations | 1.54 (0.97-2.44) | 0.065 | **2.16 (1.53-3.07)** | 0.000 | **2.30 (1.50-3.54)** | 0.000 | **2.36 (1.72-3.24)** | 0.000 |
| Antiepileptics | **2.05 (1.35-3.11)** | 0.001 | **3.42 (2.51-4.66)** | 0.000 | **2.55 (1.79-3.63)** | 0.000 | **3.44 (2.58-4.60)** | 0.000 |
| Antigout preparations | **2.30 (1.05-5.02)** | 0.037 | **1.98 (0.84-4.69)** | 0.120 | 1.72 (0.80-3.72) | 0.166 | 2.02 (0.92-4.45) | 0.082 |
| Antithrombotic agents | **1.63 (1.23-2.17)** | 0.001 | **1.94 (1.40-2.69)** | 0.000 | **1.55 (1.10-2.18)** | 0.012 | **2.48 (1.8-13.4)** | 0.000 |
| Beta blocking agents | 1.04 (0.73-1.48) | 0.830 | **1.53 (1.05-2.24)** | 0.028 | **1.69 (1.20-2.38)** | 0.002 | **1.89 (1.43-2.48)** | 0.000 |
| Calcium channel blockers | 1.41 (0.93-2.13) | 0.101 | **2.37 (1.74-3.21)** | 0.000 | **1.98 (1.33-2.95)** | 0.001 | **2.30 (1.61-3.27)** | 0.000 |
| Cardiac therapy | **2.12 (1.45-3.11)** | 0.000 | **2.41 (1.69-3.44)** | 0.000 | **2.73 (1.87-4.00)** | 0.000 | **3.48 (2.54-4.76)** | 0.000 |
| Corticosteroids for systemic use | **1.90 (1.05-3.43)** | 0.033 | 1.37 (0.62-3.02) | 0.435 | 1.60 (0.90-2.85) | 0.108 | **2.06 (1.21-3.52)** | 0.008 |
| Diuretics | **1.58 (1.13-2.19)** | 0.007 | **1.92 (1.31-2.82)** | 0.001 | **1.78 (1.18-2.69)** | 0.006 | **3.02 (2.17-4.19)** | 0.000 |
| Drugs for acid related disorders | **1.44 (1.04-1.99)** | 0.027 | **2.84 (1.99-4.05)** | 0.000 | **1.97 (1.48-2.61)** | 0.000 | **2.62 (2.01-3.41)** | 0.000 |
| Drugs for constipation | **1.52 (1.20-1.92)** | 0.001 | **2.83 (1.87-4.27)** | 0.000 | **1.82 (1.36-2.45)** | 0.000 | **2.45 (1.85-3.24)** | 0.000 |
| Drugs for obstructive airway diseases | **1.82 (1.07-3.09)** | 0.028 | **1.80 (1.04-3.12)** | 0.037 | **2.25 (1.30-3.88)** | 0.004 | **2.70 (1.58-4.62)** | 0.000 |
| Drugs used in diabetes | **1.78 (1.14-2.76)** | 0.011 | 1.69 (0.99-2.87) | 0.055 | **2.02 (1.16-3.50)** | 0.013 | 1.48 (0.90-2.44) | 0.119 |
| Lipid modifying agents | **1.51 (1.10-2.07)** | 0.010 | **1.99 (1.34-2.95)** | 0.001 | **2.17 (1.57-2.98)** | 0.000 | **2.82 (2.00-3.96)** | 0.000 |
| Mineral supplements | **1.56 (1.19-2.07)** | 0.002 | **2.80 (2.13-3.67)** | 0.000 | **1.55 (1.12-2.15)** | 0.009 | **2.73 (2.17-3.44)** | 0.000 |
| Ophthalmologicals | **1.89 (1.33-2.69)** | 0.000 | **3.07 (2.27-4.16)** | 0.000 | **2.47 (1.62-3.78)** | 0.000 | **3.55 (2.68-4.70)** | 0.000 |
| Psychoanaleptics | 1.08 (0.80-1.47) | 0.604 | **1.77 (1.30-2.43)** | 0.000 | **1.65 (1.17-2.31)** | 0.004 | **1.95 (1.43-2.67)** | 0.000 |
| Psycholeptics | **1.45 (1.07-1.97)** | 0.016 | **2.15 (1.44-3.22)** | 0.000 | **1.57 (1.09-2.27)** | 0.016 | **3.23 (2.39-4.35)** | 0.000 |
| Thyroid therapy | 0.87 (0.52-1.46) | 0.598 | 1.38 (0.95-2.02) | 0.092 | 1.29 (0.85-1.96) | 0.236 | **1.52 (1.00-2.30** | 0.048 |
| Urologicals | 1.63 (0.98-2.70) | 0.059 | **2.26 (1.50-3.39)** | 0.000 | **1.96 (1.18-3.25)** | 0.009 | **3.13 (2.21-4.44)** | 0.000 |
| Vitamins | 1.24 (0.97-1.58) | 0.088 | **1.67 (1.11-2.52)** | 0.014 | **1.70 (1.28-2.24)** | 0.000 | **2.77 (1.98-3.87)** | 0.000 |

*****Peptic Ulcer Disease & Gastro-Oesophageal Reflux Disease


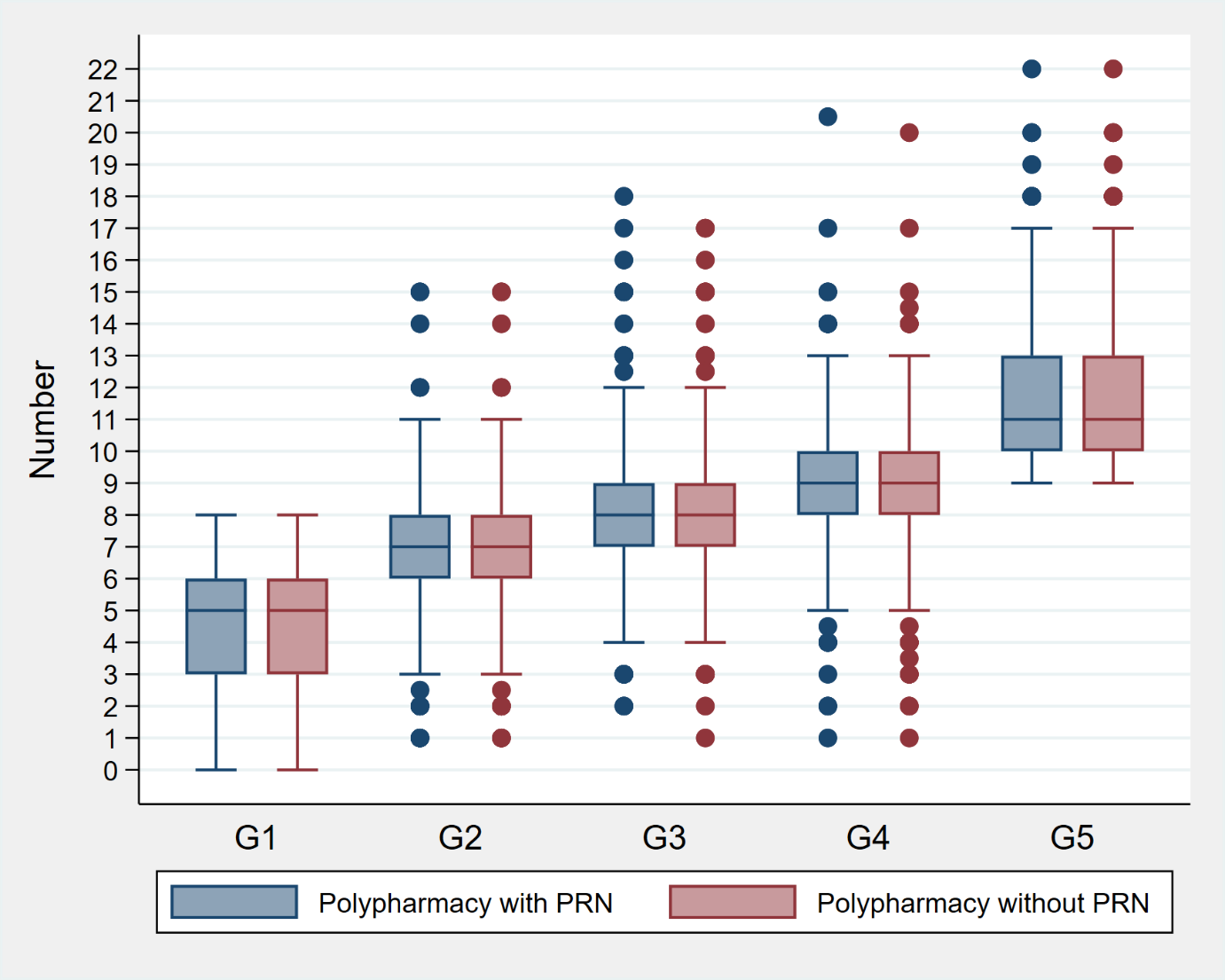


**Supplementary Figure 1: Median number of medications by trajectory groups:** 5 (IQR 3-6; range 0-8) for group 1; 7 (IQR 6-8; range 1-15) for group 2; 8 (IQR 7-9; range 1-17) for group 3; 9 (IQR 8-10; range 1-20) for group 4 and 11 IQR 10-13; range 9-22) for group 5.
